# Medical Teaching in Louisville, Kentucky

**Published:** 1883-10

**Authors:** 


### MEDICAL TEACHING IN LOUISVILLE, KENTUCKY.

The business of medical teaching is overdone in Louisville. Our counsel under the present tangle of the situation is to call a convention of all concerned and merge the two summer and the two winter schools into one first class winter school. Such an arrangement will be good for Kentucky, for Louisville, for the entire medical profession South and West.

The truth is, there is too much medical professional ambition of a wrong sort in Louisville. Let every tenth aspirant for a college professorship be put upon the retired list, and if he will not be retired, let him be decently shot. We commend this to the struggling brethren there as the best thing to be done under the circumstances.

